# Is liquid biopsy the future commutator of decision-making in liver transplantation for hepatocellular carcinoma?

**DOI:** 10.3389/fonc.2022.940473

**Published:** 2022-08-11

**Authors:** Stéphanie Gonvers, Parissa Tabrizian, Emmanuel Melloul, Olivier Dormond, Myron Schwartz, Nicolas Demartines, Ismail Labgaa

**Affiliations:** ^1^ Department of Visceral Surgery, Lausanne University Hospital (CHUV), University of Lausanne (UNIL), Lausanne, Switzerland; ^2^ Recanati Miller Transplant Institute, The Icahn School of Medicine at Mount Sinai Hospital, New York, NY, United States; ^3^ Mount Sinai Liver Cancer Program, Division of Liver Diseases, Tisch Cancer Institute, Icahn School of Medicine at Mount Sinai, New York, NY, United States

**Keywords:** CTC (circulation tumor cells), ctDNA (circulating tumor DNA), liver cancer, transplant, biomarkers

## Abstract

Liver transplant (LT) is the most favorable treatment option for patients with early stage hepatocellular carcinoma (HCC). Numerous attempts have been pursued to establish eligibility criteria and select HCC patients for LT, leading to various systems that essentially integrate clinico-morphological variables. Lacking of sufficient granularity to recapitulate the biological complexity of the disease, all these alternatives display substantial limitations and are thus undeniably imperfect. Liquid biopsy, defined as the molecular analysis of circulating analytes released by a cancer into the bloodstream, was revealed as an incomparable tool in the management of cancers, including HCC. It appears as an ideal candidate to refine selection criteria of LT in HCC. The present comprehensive review analyzed the available literature on this topic. Data in the field, however, remain scarce with only 17 studies. Although rare, these studies provided important and encouraging findings highlighting notable prognostic values and supporting the contribution of liquid biopsy in this specific clinical scenario. These results underpinned the critical and urgent need to intensify and accelerate research on liquid biopsy, in order to determine whether and how liquid biopsy may be integrated in the decision-making of LT in HCC.

## Background

Primary liver cancer and its main form hepatocellular carcinoma (HCC) are estimated to result in over 1 million of deaths by 2030 (1). A majority of new cases are unfortunately diagnosed at advanced stages with dismal outcomes, but a future shift of this paradigm may be expected, leading to an increasing number of patients diagnosed at early stage because of new measures applied to improve surveillance. Patients with early stage HCC may receive curative treatments, being essentially surgery with either liver resection (LR) or liver transplant (LT), according to liver function. LT represents an ideal option combining the advantages of removing both tumor and cirrhotic liver which is associated with a risk of *de novo* HCC occurrence; this treatment is thereby associated with the best outcomes, with 5-year survival rates reaching 70%–80% (2–4). LT for HCC is a challenging clinical scenario with patients having at least two major diseases (i.e., cirrhosis and cancer) where the maxim “*primum non nocere, secundum cavere et tertium sanare*” particularly sounds. The main stake resides in the selection process, aiming to not only avoid selecting patients who will not benefit from LT but also preventing from excluding the ones who may benefit from a new liver. This decision is complexified by several considerations, among them: lifelong immunosuppressive treatment needed after LT, therapeutical options to treat HCC recurrence after LT remain limited with poor outcomes, and, importantly, the worldwide dramatic organ shortage. Tremendous efforts have been pursued to delineate the eligibility criteria of LT for HCC. In 1996, Mazzaferro et al. published an algorithm based on radio-morphologic variables, revealed as the backbone of decision-making in LT for HCC (5). Later, this system has been criticized and challenged by numerous alternatives that followed different approaches: based on morphometrics (6–8), also integrating biological factors like alpha-fetoprotein (AFP) or prothrombin induced by vitamin K antagonist-II (PIVKA-II) (4, 9) or even using new technologies like artificial intelligence (10). Regardless of the strategy, those algorithms primarily relied on clinical variables and eventually included AFP or PIVKA-II but lacked of molecular biomarkers capable of recapitulating the biological complexity of HCC. Studies investigating the potential contribution of molecular markers remain scarce and mostly derived from tissue-based biomarkers (11).

Liquid biopsy is defined as the molecular analysis of tumor by-products released into the bloodstream by solid cancers and has shown very promising results, including in HCC (12). Only requiring simple blood tests, it represents an incomparable niche for biomarkers discovery. LT for HCC is a typical clinical context where liquid biopsy may be highly valuable, allowing to identify biomarkers offering higher granularity and better prognostic value for long-term outcomes (13). It appears as an ideal strategy to improve decision-making in LT for HCC.

The present review aims to summarize the available data on liquid biopsy in patients undergoing LT for HCC for each circulating analytes and to discuss the perspectives of this approach.

## Liquid biopsy in liver transplant for hepatocellular carcinoma

**Figure 1:**
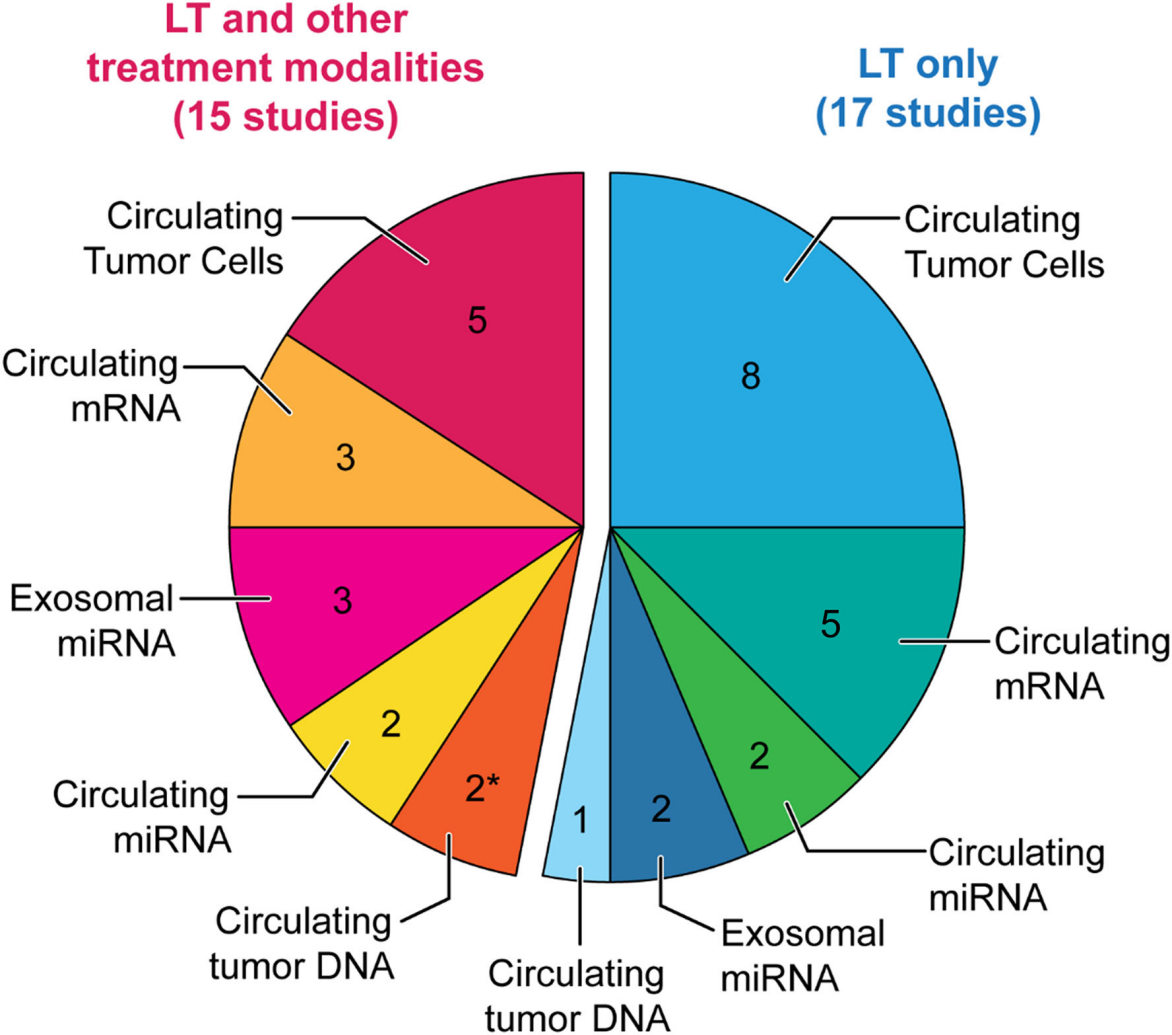
Pie chart of the available studies on liquid biopsy in LT for HCC. Pie chart illustrating the number of available studies on liquid biopsy in liver transplant (LT) for hepatocellular carcinoma (HCC), for each circulating analyte. Blue: Studies only including LT. Pink: Studies including LT and other treatment modalities. * including one study providing preliminary data (14).

**Figure 1** summarizes the available studies on liquid biopsy in HCC patients undergoing LT. Briefly, a total of 32 studies were identified, but only 17 were specifically dedicated to LT, whereas 15 studies also included other treatment modalities. The review will primarily focus on studies including only LT, as the other studies mostly lacked specific conclusions.

## Circulating tumor cells

The biological mechanisms driving metastatic diseases have been depictured by the “seed and soil” theory where seeds would represent malignant cells capable of detaching from the primary tumor and circulate toward distant organs where they engraft, proliferate and form new malignant nodules, namely metastases. These cells have been referred as circulating tumor cells (CTCs). Unsurprisingly, they suscitated a strong interest in cancer research and became the target of numerous investigations. The majority of these studies assessed the prognostic value of CTCs for recurrence and/or survival. As an example, Cristofanilli et al. analyzed the impact of CTCs on outcomes in patients with metastatic breast cancer (15). The study showed that patients above the determined threshold of five detected CTCs/7.5 ml of blood had poorer outcomes, with shorter median progression-free survival (PFS) (2.7 months vs. 7.0 months, *p* < 0.001) and shorter overall survival (OS) (10.1 months vs. >18 months, *p* < 0.001). In addition, on multivariable analysis, CTCs count was identified as the most significant prognostic factor for PFS and OS.

Likewise, CTCs were also investigated in HCC, with studies displaying a *crescendo* sophistication overtime: The first challenge was to demonstrate the feasibility of detecting CTCs in HCC patients. Thereafter, studies enumerated CTCs and analyzed the prognostic value of detected CTCs count (16, 17). Finally, studies even characterized these CTCs on a molecular level, with single-cell sequencing (18).

### Circulating tumor cells in liver transplant at a glance

There is a limited amount of data on CTCs in HCC, where most studies were conducted in patients undergoing LR. The data deriving from patients undergoing LT for HCC are even scanter. **Table 1** summarizes the selected studies. Among 13 articles, only eight studies specifically focused on LT (19–26) (**Table 1**), including only transplant patients, whereas five other studies investigated CTCs in cohorts of patients receiving various treatments (27–31).

**Table 1:** Circulating tumor cells in liver transplantation for hepatocellular carcinoma.

Treatment (Number of patients)	Approach	Technique(s)	Markers	Threshold	Time points	Main finding(s)	Refs
**LT (21)**	Enumeration	CellSearch^®^ IsoFlux^®^ and immunofluo-rescence staining	CK8^+^ CK18^+^ CK19^+^ DAPI^+^ CD45^−^	≥ 2/7.5 ml	Pre-LT	Comparison of detection performance of IsoFlux^®^ vs. CellSearch^®^: IsoFlux^®^ detected CTCs in 90.5% patients compared to 4.7% for CellSearch^®^ (*p* < 0.05). Prognostic value of CTCs was not assessed.	(19)
**LT (30)** HC (10)	Enumeration	iFISH^®^ CellSearch^®^	CEP8(≥2) CK^+^ DAPI^+^ CD45^−^	> 5/7.5 ml	Pre-LT Post-LT (3 months)	Comparison of detection performance of iFISH^®^ vs. CellSearch^®^: performance of iFISH^®^ was higher than CellSearch^®^ (sensitivity 70% vs. 26.7%; *p* < 0.01). Pre-LT iFISH^®^ CTC count predicted recurrence on univariable analysis (HR, 5.14; 95% CI, 1.53-17.31; *p* = 0.008).	(20)
**LT (24)**	Enumeration	IsoFlux^®^ and immunofluores-cence staining	CK^+^ DAPI^+^ CD45^−^	≥ 1/10 ml	Pre-LT Post-LT (1 month and 6 months)	Pre-LT CTC count correlated with time spent on the waiting-list for LT (ρ = 0.413; *p* = 0.04). Prognostic value of CTCs was not assessed.	(21)
**LT (193)**	Enumeration Characterization	ChimeraX^®^-i120 Single-cell whole genome sequencing (WGS, n=3)	EpCAM^+^ Pan-CK^+^ CK19^+^ DAPI^+^ CD45^−^	≥ 1/5 ml	Pre-LT Post-LT (1 month and months)	Pre-LT CTC count showed low predictive value for recurrence. Post-LT CTC count was a prognostic factor for recurrence (HR, 2.67; 95% CI, 1.51–4.74; *p* = 0.001).	(22)
**LT (50)**	Enumeration	Negative enrichment and imFISH	CEP8(≥ 3) DAPI^+^ CD45^−^	> 1/3.2 ml	Pre-LT	Pre-LT CTC count was a prognostic factor for recurrence (RR, 5.41; 95% CI, 1.13–25.87; *p* = 0.034).	(23)
**LT (47)**	Enumeration Characterization	CanPatrol™ and RNA-ISH	EpCAM^+^ CK8^+^ CK18^+^ CK19^+^ DAPI^+^ CD45^−^ Vimentin+ Twist^+^	≥ 2/5 ml	Pre-LT Post-LT (1 month and months)	Three different subtypes of CTCs were identified: epithelial, interstitial and mixed. Post-LT, changes in the proportion of CTCs subtypes were observed (increased epithelial and interstitial CTC levels). CTC count was not associated with recurrence (*p* > 0.05).	(24)
**LT (56)**	Enumeration Characterization	CanPatrol™ and RNA-ISH	EpCAM^+^ CK8^+^ CK18^+^ CK19^+^ Vimentin^+^ Twist^+^	≥ 1/5 ml	Pre-LT Post-LT (POD7-10)	Three different subtypes of CTCs were identified: epithelial, interstitial and mixed. Interstitial CTCs showed particular interest. A perioperative increasing proportion of interstitial CTC was a prognostic factor of recurrence (HR, 6.17; 95% CI, 1.89–20.18; *p* = 0.003).	(25)
**LT (25)**	Enumeration Characterization	Fluorescence-activated cell sorting (FACS Calibur)	EpCAM^+^ CD90^+^ CD45^−^	≥ 1/10 ml	Pre-LT Post-LT (POD1/7)	Three different subtypes of CTCs were identified: EpCAM^+^ (epithelial), CD90^+^ (mesenchymal) and EpCAM^+^/CD90^+^ (mixed). Pre-LT, EpCAM^+^ CTC count was associated with lower DFS (*p* = 0.025). Detection of EpCAM^+^/CD90^+^ CTCs on POD 1 was a prognostic factor of recurrence (HR, 26.88; 95% CI, 1.86–387.51; *p* = 0.016).	(26)

LT, liver transplant; CK, cytokeratin; DAPI, 4′, 6-diamino-2-fenilidol; CD, cluster of differentiation; CTC, circulating tumor cell; HC, healthy controls; iFISH, interphase fluorescence in situ hybridization; CEP8, fluorescent labeled DNA probe specific for the centromeric region of chromosome 8; HR, hazard ratio; CI, confidence interval; p, p-value; EpCAM, epithelial cell adhesion molecule; imFISH, immunofluorescence in situ hybridization; RR, relative risk; RNA-ISH, ribonucleic acid in situ hybridization; Vimentin and Twist, mesenchymal biomarkers; POD, postoperative day; DFS, disease-free survival.

### Circulating tumor cells detection in liver transplant: Techniques, kinetics, and correlations

The most widely used and only Food and Drug Administration (FDA)–approved system for CTCs detection is CellSearch^®^, an antibody-based platform targeting epithelial cell adhesion molecule (EpCAM) positive circulating cells. In HCC, this approach has been repeatedly questioned, since only around 30% of HCC cells express EpCAM (25, 28). Therefore, it may not be the best strategy to detect CTCs in HCC and studies compared CellSearch^®^ with other technologies. In 2015, a study compared CellSearch^®^ with IsoFlux^®^ in a cohort of 21 patients undergoing LT, showing a drastic difference of CTCs isolation between the two systems (4.7% of patients for CellSearch^®^ vs. 90.5% for IsoFlux^®^); prognosis was not analyzed in this article (19). A similar study compared CellSearch^®^ with iFISH^®^ in a cohort of 30 HCC patients and 10 healthy controls. Again, CellSearch^®^ showed a low sensitivity of 26.7% compared with 70% for iFISH^®^. CTCs detected by iFISH^®^, with a threshold of ≥ 5 CTCs, was a factor associated with shorter PFS on univariable analysis but the report lacked multivariable analysis (20). Recently, Amado et al. studied the kinetics of CTCs clearance after LR and LT, utilizing iFISH^®^ and collecting sequential blood samples pre-operatively, on post-operative day (POD) 5 and POD30 (27). Despite a comparable preoperative CTCs count, LT was followed by a significant proportion of CTCs clearance (*p* = 0.007), conversely to LR (*p* = 0.241). Moreover, the detection of clusters—defined as ≥ 3 aggregated cells—was associated with an increased risk of incomplete clearance on POD30, which was identified as a prognostic factor of shorter OS (*p* = 0.038) in the whole cohort. Another study also used IsoFlux^®^ to enumerate CTCs in 24 HCC patients within the waiting list for LT; authors performed correlation analyzes between CTCs and AFP, as well as PET-CT values (21). No correlation between these markers was detected, but CTCs count positively correlated with the time spent on the waiting list (ρ = 0.413, *p* = 0.04), despite a persistent compliance to Milan criteria.

### The prognostic value of circulating tumor cells in liver transplant

Recently, Wang et al. published the largest study on HCC–CTCs in LT with a cohort of 193 patients, aiming to assess the predictive value of CTCs on recurrence (22). On multivariable analysis, the post-operative detection of CTCs was the most significant prognostic factor of recurrence (hazard ratio [HR], 2.67; 95% confidence interval [CI], 1.51–4.74; *p* = 0.001), outperforming variables such as tumor size, number, vascular invasion, and AFP level. However, the prognostic value of pre-operative CTCs count showed low area under curve (AUC) values, regardless of the cutoff. Pre-operative CTCs are of particular interest, as they may represent a potential criteria of eligibility to LT. Chen et al. specifically focused on preoperative CTCs in a study with 50 HCC patients undergoing LT (23). Using negative enrichment with CD45- and iFISH^®^, CTCs were detected in 26 (52%) patients and CTCs count positively correlated with several other prognostic factors like tumor size (χ^2 ^= 5.77, *p* = 0.016), AFP level (χ^2 =^ 5.45, *p* = 0.02) and tumor grade (χ^2 =^ 6.48, *p* = 0.039). Furthermore, it was the only prognostic factor of recurrence identified by multivariable analysis (relative risk [RR], 5.41; 95% CI, 1.13–25.87; *p* = 0.034).

The following studies sought to identify different CTCs subtypes and thereby added an additional layer of information; these three studies identified three different HCC–CTCs subtypes based on cell surface markers: epithelial, interstitial or mesenchymal, and mixed. In a cohort of 47 patients, CTCs subtypes showed a perioperative fluctuation with increasing levels of epithelial and interstitial subtypes after LT (24). Nevertheless, CTCs count showed no association with recurrence, neither for the total count nor for any subtypes. Identifying the same CTCs subtypes, another study with 56 patients, showed that a perioperative increase of intestinal CTCs was an independent factor of recurrence (25). An interesting finding of this study was to highlight the value of ΔCTCs. In other words, results showed that the post-operative count of interstitial CTCs alone was not an independent factor of recurrence (RR, 4.04; 95% CI, 0.92–17.70; *p* = 0.064), whereas the perioperative fluctuation of this CTCs-subtype was a prognostic factor (RR, 6.17; 95% CI, 1.89–20.18; *p* = 0.003). This illustrates the priceless advantage of liquid biopsy, allowing to easily repeat blood samples and analyses that result in dynamic markers capable of reflecting the course of the disease and capturing its significance. In a cohort of 25 HCC patients undergoing living donor liver transplantation (LDLT), three subtypes of CTCs were detected: epithelial (EpCAM^+^), mesenchymal (CD90^+^) and mixed (EpCAM^+^/CD90^+^) (26). Multivariable analysis of recurrence identified two independent factors: pre-LT serum PIVKA-II ≥100 mAU/ml (HR, 14.64; 95% CI, 1.08–198.20; *p* = 0.043) and the detection of mixed CTCs on POD1 (HR, 26.88; 95% CI, 1.86–387.51; *p* = 0.016).

## Circulating mRNAs

Unlike miRNAs that are relatively stable in plasma and exosomal RNAs that are protected in a micro-vesicle, circulating mRNAs are instable and thus difficult to study. Data in HCC are scarce and mainly focused on albumin and AFP.

### Circulating mRNAs in liver transplant at a glance

A total of eight studies investigating circulating mRNAs in LT for HCC were selected, with five studies focusing on LT (26, 32–35) (**Table 2**), whereas three other studies included various treatments and brought no conclusion specific to LT (38–40).

**Table 2:** Circulating mRNAs and miRNAs in liver transplantation for hepatocellular carcinoma.

Treatment (Number of patients)	Readout(s)	Technique(s)	Time-point(s)	Main finding(s)	Refs
**Circulating mRNA (5 studies)**					
**LT** (82): **- HCC (72)** - CLD (10) HC (10)	Albumin	qRT-PCR	Pre-LT	Pre-LT, high level of albumin mRNA was a prognostic factor of: - Recurrence (HR, 5.9; 95% CI, 1.9–18.8; *p* = 0.002) - OS (HR, 4.6; 95% CI, 1.6–13.8; *p* = 0.006) - RFS (HR, 4.3; 95% CI, 1.6–11.8; *p* = 0.005).	(32)
**LT (14)**	• h-TERT • AFP	RT-PCR	• Pre-LT • Post-LT	Pre-LT h-TERT mRNA level was associated with RFS (*p* = 0.005) but not AFP mRNA (*p* = 0.23).	(33)
**LT** (48): **- HCC (32)** - ESLD (16) LDC (48)	AFP	qRT-PCR	• Pre-LT • Intra-LT • Post-LT	Pre-LT AFP mRNA was a prognostic factor of recurrence (HR, 10.8; 95% CI, 1.53–76.9; *p* = 0.017).	(34)
**LT** (49): **- HCC (29)** - CLD (20) HC (20)	• AFP • GPC3	RT-PCR	• Pre-LT • Intra-LT • Post-LT	• Pre-LT, AFP mRNA level was a prognostic factor of recurrence (RR, 2.91; 95% CI, 1.09–7.76; *p* = 0.033). • Post-LT, AFP mRNA level was not a prognostic factor of recurrence (RR, 2.62; 95% CI, 0.93–7.41; *p* = 0.07). • GPC3 mRNA level was not associated with recurrence.	(35)
**LT (25)**	• K19 • EpCAM • CD90 • SNAIL • TWIST	qRT-PCR	• Pre-LT • Post-LT (POD 1/7)	• EpCAM and CD90 mRNA levels correlated with the detection rate of EpCAM^+^ and CD90^+^ CTCs but showed no prognostic value. • mRNA levels of K19, SNAIL and TWIST were not associated with recurrence.	(26)
Circulating miRNA (2 studies)					
**LT** (213): **- HCC (193)** - ESLD (20)	• miR-122 • miR-192 • miR-21 • miR-223 • miR-26a • miR-27a • miR-801	qRT-PCR	• Pre-LT • Post-LT (POD1-6/7–14)	• Positive mi-R panel status in the late phase (7–14 days) was a prognostic factor of recurrence (HR, 4.90; 95% CI, 2.20–10.95; *p* < 0.001). • mi-R panel was an earlier predictor of recurrence than AFP and DCP. In addition, it preceded evidence of recurrence on imaging with a median delay of 2.4 months.	(36)
**LT (62)** HC (12)	• miR-148a • miR-1246 • miR-1290 • Let7c • miR-21 • miR-23b • miR-27b • miR-122 • miR-125b • miR-151-5p • miR-192 • miR-195 • miR-199a-3p • miR-215	• microarray profiling • qRT-PCR	• Pre LT • Post-LT (2h, POD1 and 1w)	In the early phase (2-h after portal vein reperfusion), upregulation of miR-1246 was a prognostic predictor of both DFS (HR, 10.12; 95% CI, 1.45–70.47; *p* = 0.020) and OS (HR, 10.24; 95% CI, 1.39–75.67; *p* = 0.023).	(37)

LT, liver transplantation; HCC, hepatocellular carcinoma; CLD, chronic liver disease; HC, healthy controls; qRT-PCR, real-time quantitative reverse transcription polymerase chain reaction; HR, hazard ratio; CI, confidence interval; p, p-value; OS, overall survival; RFS, recurrence-free survival; h-TERT, human-telomerase reverse transcriptase; AFP, alpha-fetoprotein; ESLD, end-stage liver disease; LDC, live donors (control group); GPC3, glypican-3; RR, relative risk; K19, keratin 19; EpCAM, epithelial cell adhesion molecule; CD90, cluster of differentiation 90; SNAIL and TWIST, epithelial-mesenchymal transition (EMT)–related genes; DCP, des-gamma-carboxyprothombin; DFS, disease-free survival.

### The prognostic value of circulating mRNAs in liver transplant

The level of albumin mRNA was measured in plasma samples of 72 HCC patients undergoing LT (32). Patients were dichotomized in low versus high albumin mRNA level, and this variable was tested in cox regression models for recurrence, OS, and recurrence-free survival (RFS). Consistently, vascular invasion and high plasma albumin mRNA level were the only independent prognostic factors of each endpoints listed above. In a pilot study, levels of *h-TERT* (human telomerase reverse transcriptase) and AFP mRNAs were examined in 14 patients (33). Multivariable analysis was not performed, probably because of the small sample size. Nevertheless, patients with positive h-TERT mRNA showed lower RFS (*p* = 0.005), whereas no association with AFP mRNA was detected. Conversely, two other studies highlighted the potential prognostic value of AFP mRNA. Marubashi et al. tested its role in a cohort of 32 HCC undergoing LDLT (34). Positive preoperative plasma AFP mRNA was an independent predictor of recurrence (HR, 10.8; 95% CI, 1.53–76.9; *p* = 0.017). Results were similar in another study confirming the prognostic value of AFP but failing to demonstrate the value of GPC3 (35). In their study already discussed within the CTCs section, Hwang et al. also analyzed circulating mRNAs (26). Although mRNA levels of EpCAM and CD90 correlated with the detection rates of EpCAM+ and CD90+ CTCs, they showed no prognostic value. Likewise, mRNA levels of K19, SNAIL and TWIST were not associated with outcomes.

## Circulating miRNAs

MiRNAs have been extensively studied in cancers including HCC, but mostly in tissue samples. As they are pretty stable in plasma, circulating miRNAs also became the center of attention in different research groups. As an example, a prospective study underpinned the potential contribution of miRNAs-based liquid biopsy for HCC surveillance in cohorts of patients at risk, outperforming AFP (41).

### Circulating miRNAs in liver transplant at a glance

Despite its attractive characteristics, circulating miRNAs was rarely investigated in LT, with a total of four studies: two focusing on LT (36, 37) (**Table 2**) and two studies also including other treatments (42, 43).

### The prognostic value of circulating miRNAs in liver transplant

Huang et al. designed a panel of seven circulating miRNAs (miR-122, miR-192, miR-21, miR-223, miR-26a, miR-27a, and miR-801), which was tested preoperatively, 1–6 days (early phase) and 7–14 days (late phase) after LT (36). As first finding, positive miRNA panel status at late phase was the only independent prognostic factor of recurrence (HR, 4.90; 95% CI, 2.20–10.95; *p* < 0.001). In addition, the results underscored the value of this panel as early predictor allowing to literally anticipate recurrence: the dynamic monitoring of this panel showed that a change from negative to positive status preceded AFP and Des-Gamma-Carboxyprothrombin (DCP) as well as the radiological evidence of relapse, with a median interval of 2.4 months.

A comprehensive study selected 14 circulating miRNAs showing differential profiles between patients with and without recurrence, subsequently validated in 62 HCC patients undergoing LT (37). Association between endpoints and candidates (miR-148a, miR-1246, and miR-1290) were identified but miR-1246 in the early phase (2h after portal vein re-perfusion) was the most pertinent biomarker, being the only independent prognostic factor of both DFS (HR, 10.12; 95% CI, 1.45–70.47; *p* = 0.02) and OS (HR, 10.24; 95% CI, 1.39–75.67; *p* = 0.023).

## Exosomal RNAs

Exosomes are members of the family of extracellular vesicles (EVs); more precisely, they are defined as small EVs (44). They contain a variety of cargo such as DNA and RNA fragments, which are protected from degradation in plasma. Their roles is getting increasingly elucidated and seem to include a wide range of contributions, especially inter-cellular communication (45–47). The data on exosomes in HCC remain modest, but recent studies highlighted their value for surveillance (48) and prognostication (49). Of note, large EVs have also shown promises in HCC (50, 51), but specific data on LT are still needed.

### Exosomal RNAs in liver transplant

Five studies analyzed exosomes, but only two of them specifically focused on LT (49, 52–55) (**Table 3**). These two studies followed a translational approach with animal models and human samples. In a cohort of patients undergoing LDLT, HCC patients showed upregulated exosomal miR-92b before transplant (53). In addition, the post-LT level of this marker demonstrated high accuracy to predict early recurrence with an AUC of 0.925 (*p* < 0.001) yielding sensitivity and specificity of 85.7% and 86.0%, respectively. Sugimachi et al. quantified expression profiling of exosomal miR in six HCC patients and were able to distinguish miR differentially expressed in patients with and without recurrence: miR-718 and miR-1246 were down- and upregulated, respectively (54). Thereafter, this group analyzed the clinical significance of this differential expression in a validation cohort of 59 HCC patients undergoing LT. Low expression of miR-718 was associated with poor differentiation (*p* = 0.026) and a higher likelihood to be beyond Milan criteria (*p* = 0.04). Nevertheless, no association with recurrence was detected (*p* = 0.13), potentially because of the small number of events. In subgroup analysis, authors showed that patients with high level of miR-718 and tumor < 3cm had higher RFS rate than patients with low level of miR-718 and tumor ≥ 3cm (*p* = 0.002). Of note, multivariable analysis was not performed.

**Table 3:** Exosomal RNAs and circulating-free DNA in liver transplantation for hepatocellular carcinoma.

Treatment (Number of patients)	Readout(s)	Technique(s)	Time points	Main finding(s)	Refs
**Exosomal miRNA (2 studies)**					
**LT** (121): **- HCC (93)** - CLD (28)	Exosomal miR-92b	• microarray profiling • qRT-PCR	• Pre-LT • Post-LT (1 month)	Post-LT, exosomal miR-92b level predicted early recurrence (AUC = 0.925, *p* < 0.001; sensitivity = 85.7%, specificity = 86.0%).	(53)
**LT (65)**	Exosomal • miR-718 • miR-1246	• microarray profiling • qRT-PCR	Pre-LT	Exosomal miR-718 and miR-1246, were significantly downregulated and upregulated, respectively, in patients with recurrence compared to non-recurrent patients. Low expression of miR-718 was associated with poorer histological differentiation (*p* = 0.026) and beyond Milan criteria status (*p* = 0.04). Exosomal miR-718 expression level was not associated with RFS (*p* = 0.13).	(54)
**Circulating-free DNA (1 study)**					
**LT (50)**	cfDNA 90 cfDNA 222	qRT-PCR	Post-LT (immediate, POD 1/3/7)	High level of cfDNA 90 bp was the only independent prognostic factor of 1-year survival (HR, 11.96; 95% CI, 1.11–128.96; *p* = 0.041).	(56)

LT, liver transplantation; HCC, hepatocellular carcinoma; CLD, chronic liver disease; qRT-PCR, real-time quantitative reverse transcription polymerase chain reaction; AUC, area under curve; p, p-value; RFS, recurrence-free survival; POD, postoperative day; HR, hazard ratio; CI, confidence interval.

## Circulating tumor DNA

In cancer patients, circulating-free DNA (cfDNA) includes DNA fragments released by both healthy and cancer cells, the so-called circulating tumor DNA (ctDNA). During the last decades, ctDNA was revealed as an extensive family of polyvalent biomarkers with numerous applications, including in HCC (57, 58).

### CtDNA in liver transplant

The data on ctDNA in LT for HCC patients are virtual. In fact, only three studies were identified: one with very preliminary data (14), one not only specifically on LT but also including LR (59) and a third one on cfDNA but not on ctDNA (56) (**Table 3**). This study was not part of the liquid biopsy framework per se. It relied on the hypothesis that cfDNA represents an endogenous damage-associated molecular pattern (DAMP) able to trigger immune response and therefore appearing as a surrogate marker of survival after LT. Amplicons of 99 and 222 bp were targeted and quantified by real-time quantitative reverse transcription polymerase chain reaction (qRT-PCR). As first finding, high levels of cfDNA were associated with other inflammatory markers such as C-Reactive Protein (CRP), leukocytosis, and granulocytosis. In addition, it was also associated with portal hepatitis and more intense neutrophils infiltrate of the graft. Finally, high level of cfDNA fragments of 90 bp was the only prognostic factor of survival at 1 year (HR, 11.96; 95% CI, 1.11–128.96; *p* = 0.041).

## Discussion

LT for HCC is a complex domain with divergent strategies of management and several controversies. A uniquely undebated point is that there is room for improvement in the selection process of HCC patients who should be transplanted. In the present review, it was hypothesized that liquid biopsy may be a pertinent tool to reach this objective and aimed to thoroughly review the available literature on liquid biopsy in LT for HCC. This literature was disappointingly scant with only 17 available studies specific to the topic (**Figure 1**), an inconsistency considering the 6’355 results for “Liquid Biopsy” on PubMed.gov (22 April 2022). Nevertheless, these rare elements of information provided encouraging data. Overall, 10 of the 17 studies performed multivariable analysis to identify prognostic factors of recurrence and/or survival. Circulating analytes were evidenced as independent prognostic factors in 10/10 studies. In five studies, they outperformed other clinico-morphological variables, whereas the remaining five multivariable models concomitantly identified circulating analytes and clinico-morphological items as independent prognostic factors.

CTCs were the most investigated analytes with eight studies. Results were promising, as illustrated by the identification of pre-LT CTCs count as a prognostic factor of recurrence (23). These criteria may be considered to select transplant candidate but may not necessarily correlate with criteria like Milan ones. This hypothesis must, however, be challenged at least by two considerations: (I) HCC-CTCs detection remains to be harmonized as technologies showed variable performances, but comparisons in term of clinical significance are still lacking. (II) Heterogeneity of the spatial distribution of CTCs has been suggested (60), which could complexify the interpretation of CTCs-based liquid biopsies but which also supports the concept of analyzing each subgroups of CTCs, as performed by three selected studies discussed above (24–26).

Studies on circulating mRNA essentially include widely known candidates such as AFP, albumin, or h-TERT. It would be of great interest to explore other genes. Regarding albumin, it would be worth assessing whether its mutation may impact the level of circulating mRNA, as albumin is a frequently mutated gene in HCC (61, 62). The data on circulating miRNAs were surprisingly scarce but provided interesting findings, either highlighting the value of a particular biomarker such as miR-1246 (37) or of a specific panel (36). Exosomes deserve to be more extensively explored, particularly for exosomal mRNA, which may be highly contributive. Finally, the results of the present review of the literature on ctDNA was particularly disappointing; strikingly speaking, there was no study on ctDNA in LT for HCC, while this class of biomarkers has proven great input for many clinical applications in various cancers.

**Figure 2:**
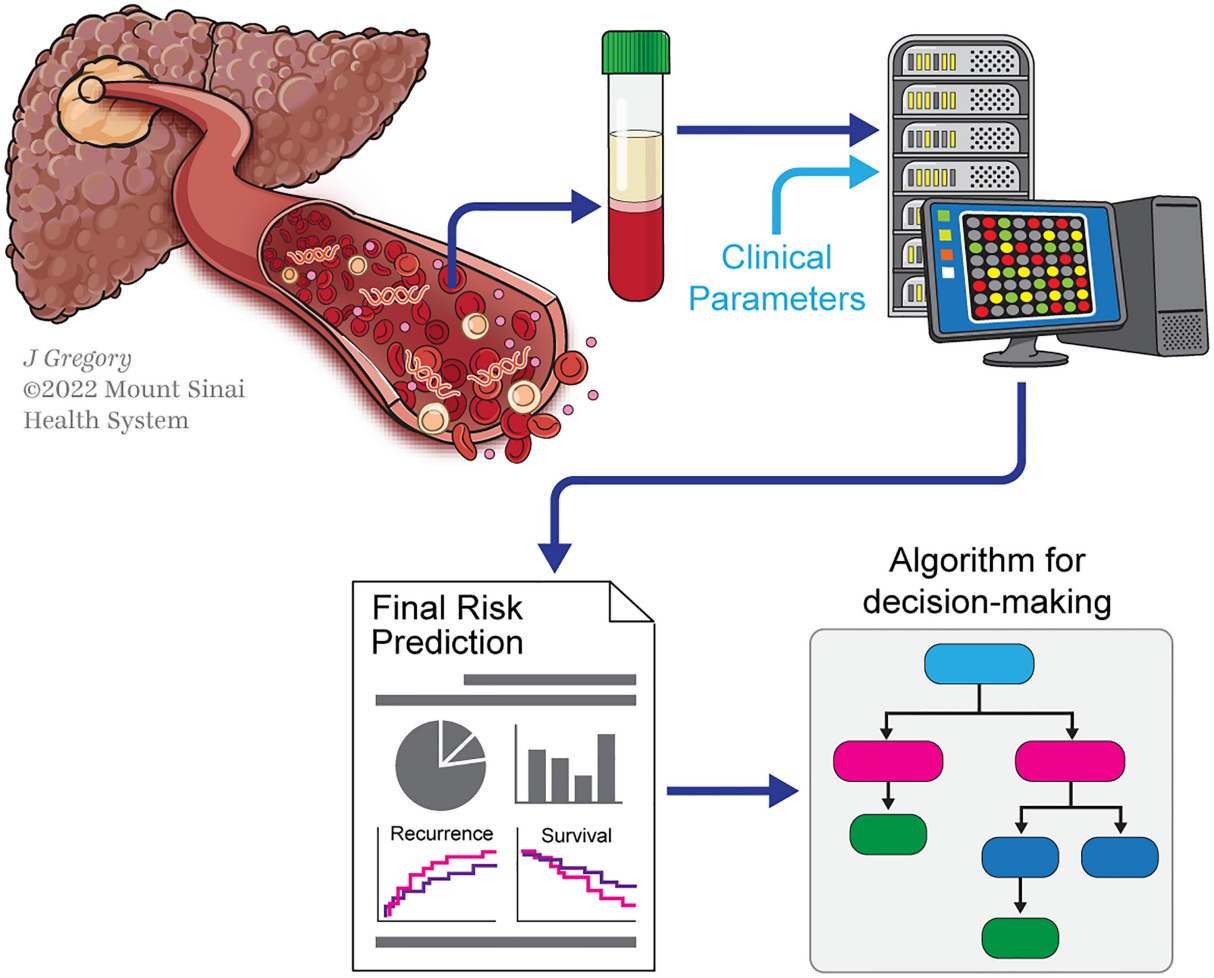
Scheme illustrating the potential contribution of liquid biopsy in decision-making for LT in HCC. Scheme illustrating a liquid-biopsy-based pipeline for decision-making in liver transplant (LT) for hepatocellular carcinoma (HCC). Collection of blood samples will allow detecting circulating analytes released by HCC into the bloodstream. Thereafter, these analytes are submitted to technologies like next-generation sequencing (NGS). The molecular analyzes of these by-products will help to determine prognosis (with outcomes such as recurrence and survival), which will ultimately guide decision-making to select patients for LT.

This study demonstrated an important gap that must be filled. Future challenges and needs especially include the intensification of research in the field, with prospective cohorts. Integrative analyzes combining different circulating analytes is also a critical unmet need. Finally, there is increasing evidence on the value of basic research with preclinical models in liquid biopsy, including in HCC (63, 64). Ultimately, these efforts will permit to establish whether liquid biopsies outperform the models currently used in clinical practice. **Figure 2** illustrates how liquid biopsy could be integrated in the decision-making of LT in HCC.

On a clinical perspective, there are several points where liquid biopsy may be helpful: (I) the main one is selection. As demonstrated, circulating analytes were identified as potent prognostic factors, frequently outperforming other confounders. Thus, it can be suggested that it may allow correcting some selection flaws. In other words, using liquid biopsy may allow on one hand to detect patients within eligibility criteria who will nonetheless develop recurrence after LT and, on the other hand, to identify patients beyond criteria such as Milan who would benefit from LT. This hypothesis is supported by data highlighting the preoperative predictive value of liquid biopsy (23, 32, 34, 35). (II) Future development of liquid biopsy in LT may probably allow subclassifying patients who may benefit from LT. This could be helpful to stratify patients and guide priority, therewith allowing to better manage patients on the waiting lists. (III) Also, as for any other biomarkers, a perfect performance cannot be expected. Some of the selected patients may nevertheless develop recurrence, but sequential blood testing will help detecting early relapse; this is supported by studies demonstrating the prognostic value of post-LT blood samples analyzes (22, 36, 37, 56). (IV) Finally, there has been increasing data demonstrating the value of downstaging strategies to optimize patients’ selection and reduce the risk of recurrence after LT (65). Although it showed a positive impact on survival, analyzes of the explants also revealed an important proportion of understaging (66). Liquid biopsy could be a reliable tool to circumvent this problem.

Of great importance, the present review focused on HCC, but the concept is likely applicable to other cancers that also became indications of LT, like cholangiocarcinoma (67) or colorectal liver metastases (CRLMs) (68). The debate for these malignancies is even more intense and the room for improvement more consequent. Liquid biopsy could be a game changer in this field too.

In conclusion, data on liquid biopsy in HCC patients undergoing LT are scarce. However, the rare available studies showed relevant and very encouraging data, supporting the value of liquid biopsy with circulating analytes of good prognostic value. There is a need to intensify research in the field in order to determine whether and how liquid biopsy may be integrated in clinical management of LT for HCC.

## Search strategy

The review of the literature was conducted *via* PubMed, using the following keywords: “liquid biopsy” AND “hepatocellular,” “transplant” AND “circulating,” “ctDNA” AND “CTC,” or “miRNA” AND “mRNA” AND “exosomes.” The search was limited to full-text articles published in English. Cross-referencing of the bibliographies from the eligible articles was also performed.

## Author contributions

Study concept and design: SG, PT, EM, OD, MS, ND, and IL; Acquisition of data: SG and IL; Analysis and interpretation of data: SG, PT, EM, MS, and IL; Drafting of the manuscript: SG and IL; Critical revision of the manuscript for important intellectual content: SG, EM, PT, MS, OD, ND, and IL. All authors contributed to the article and approved the submitted version.

## Funding

Open access funding was provided by the University of Lausanne.

## Acknowledgments

The authors would like to thank Jill Gregory for the design of **Figures 1**, **2**.

## Conflict of interest

The authors declare that the research was conducted in the absence of any commercial or financial relationships that could be construed as a potential conflict of interest.

## Publisher’s note

All claims expressed in this article are solely those of the authors and do not necessarily represent those of their affiliated organizations, or those of the publisher, the editors and the reviewers. Any product that may be evaluated in this article, or claim that may be made by its manufacturer, is not guaranteed or endorsed by the publisher.
