# Erratum: Is liquid biopsy the future commutator of decision-making in liver transplantation for hepatocellular carcinoma?

**DOI:** 10.3389/fonc.2023.1180615

**Published:** 2023-03-17

**Authors:** Frontiers Production Office

**Affiliations:** Frontiers Media SA, Lausanne, Switzerland

**Keywords:** CTC (circulation tumor cells), ctDNA (circulating tumor DNA), liver cancer, transplant, biomarkers

An omission to the funding section of the original article was made in error. The following sentence has been added: “Open access funding was provided by the University of Lausanne”.

The original version of this article has been updated.

